# Major adverse foot events and functional mobility deficits associated with diabetic neuropathy and nephropathy

**DOI:** 10.20517/mtod.2024.02

**Published:** 2024-03-31

**Authors:** David R. Sinacore, Michael A. Jones, Paul W. Kline

**Affiliations:** 1 Department of Physical Therapy, High Point University, High Point, NC 27268, USA.; 2 Department of Orthopaedic Surgery & Rehabilitation, Wake Forest University School of Medicine, Winston-Salem, NC 27157, USA.

**Keywords:** Diabetes mellitus, chronic kidney disease, risk for diabetic foot complications, major amputation

## Abstract

**Aim:**

The purpose is to determine the risk ratios (RR) for both major adverse foot events (MAFEs) and the presence of moderate and severe functional mobility deficits in participants with diabetic peripheral neuropathy across the stages of chronic kidney disease (CKD).

**Methods:**

We studied 284 participants with diabetes mellitus, peripheral neuropathy, and CKD. MAFEs including foot fracture, ulcerations, Charcot neuropathic arthropathy (CN), osteomyelitis, and minor foot amputations were collected from foot x-ray reports in the medical records of 152 participants; functional mobility deficits were assessed in 132 participants using the modified physical performance test (mPPT). Moderate mobility deficit was categorized as mPPT scores 22–29 and severe mobility deficit as < 22. Unadjusted and adjusted (age, body weight, race, HbA1c) RR were calculated across each stage of CKD, with stage 1 CKD used as the reference group.

**Results:**

The RR for neuropathic foot fracture, CN, and diabetic foot ulceration remained consistent across CKD stages. The RR of minor amputation is greater in CKD stages 4 and 5. The RR of moderate or severe mobility deficit is greater in CKD stages 3 and 5 and in CKD stages 3, 4, and 5, respectively. An inverse association was observed between MAFE prevalence and mPPT scores across CKD stages.

**Conclusion:**

Major adverse foot events and functional mobility deficits are prevalent in individuals with DPN and diabetic kidney disease. The risks for minor foot amputation and functional mobility deficits increase as early as stage 3 CKD and increase further in stages 4 and 5.

## INTRODUCTION

Peripheral neuropathy affects millions of individuals worldwide. The lifetime prevalence of peripheral neuropathy is estimated to be 50% among individuals with diabetes mellitus and this prevalence tends to increase with the duration of diabetes^[1]^. Diabetic peripheral neuropathy (DPN) typically affects the distal extremities (feet and hands) and is, therefore, recognized as one of the incipient contributors to major adverse foot events (MAFEs) including foot fracture, ulcerations, Charcot neuropathic arthropathy (CN), osteomyelitis, and minor foot amputations or foot bone resections. MAFEs can singularly and collectively lead to major lower extremity amputation^[2–5]^, which in turn can severely compromise an individual’s functional mobility and quality of life^[6]^.

Diabetic nephropathy, a form of chronic kidney disease (CKD), is another major complication of diabetes mellitus, with a reported occurrence in 20%–50% of those with diabetes^[7,8]^. Like DPN, diabetic kidney disease progresses in severity with diabetes duration. The severity of renal compromise in diabetic kidney disease can be estimated based on the rising serum creatinine levels to estimate the glomerular filtration rate (eGFR)^[9]^ with further classification of phenotypes based on urinary albumin concentration^[10]^. The eGFR reflects the degree of renal impairment and is classified into five stages, with stage 5 signifying the most severe renal impairment [National Kidney Foundation. Kidney Disease: The Basics 2022. Available from: https://www.kidney.org/news/newsroom/fsindex#fast-facts.] As both DPN and CKD progress in severity, they combine to lead to poor clinical outcomes and progressive disability^[11,12]^.

DPN combined with diabetic kidney disease progression may be the root causes of both MAFEs and early functional mobility deficits in individuals with diabetes mellitus, though this has not heretofore been demonstrated. Therefore, the purpose of this study is to describe the frequency and relative risk for major adverse foot events and functional mobility deficits in those with DPN across the stages of CKD.

## METHODS

### MAFEs study design

Data from two separate studies were utilized for the analyses presented in this manuscript. The first study involved participants identified via a retrospective electronic medical chart review of patients in the Atrium Health Wake Forest Baptist Health System diagnosed with diabetes mellitus (DM), peripheral neuropathy, and CKD stages 1–5 from 2012–2021^[13]^. The aim was to capture at least 25 individual medical records of patients with diabetes mellitus and peripheral neuropathy in each of the five stages of CKD. Our aim of 25 records selected from each stage of CKD was based on the feasibility of time allotted for completing data collection and an estimate of the sample size required to observe a difference in the outcomes of interest between participants with stage 1 CKD and stage 5 CKD^[13]^. Moreover, the inclusion criteria called for all study participants to have undergone a minimum of one foot x-ray and a comprehensive metabolic panel (CMP). The foot x-ray aimed to detect the presence of MAFEs, while the CMP was needed to obtain the serum creatinine concentration for calculating the eGFR and determining the CKD stage. Following the search, a confidential participant identification number was assigned to the medical record number (MRN) of each patient and this information was then recorded in a REDCap database. The study protocol was reviewed and approved by Wake Forest School of Medicine institutional review board (IRB00056905I) and was exempt from obtaining participant informed consent.

### Demographics and participants

Patient demographic characteristics were extracted, including age (determined at the time of foot x-ray), weight, height, body mass index (BMI), sex, race, diabetes diagnosis (type 1 or 2), peripheral neuropathy (yes or no), and dates of both the CMP and foot radiographs.

### Comprehensive metabolic panel

The serum creatinine (Cr) concentration obtained from the Comprehensive Metabolic Panel (CMP) was then used in the CKD-Epi equation to estimate the glomerular filtration rate (eGFR)^[9]^. The eGFR was used to stage the CKD severity between stages 1–5^[11]^.

### Evidence of peripheral neuropathy

Each patient’s electronic medical record was searched for evidence of having peripheral neuropathy. It was recorded as yes/present or no/absent. Typically, the presence or absence of peripheral neuropathy was found in the Past Medical History section or, when not clear, in the actual Provider’s noted objective assessment (e.g., documented as a loss of protective sensation)^[14]^.

### Major adverse foot events

The clinician reviewer (M.A.J.) also recorded verbatim the radiologists’ reports of MAFEs from the medical record, including any documentation of foot fracture, Charcot neuroarthropathy, ulceration, and/or osteomyelitis, as well as any *minor* foot amputation (i.e., partial/complete toe amputation, partial/complete ray amputation, or transmetatarsal amputation) identified via plain film radiography. The presence of each MAFE was coded individually across participants to capture those with more than 1 MAFE. The presence of each MAFE was used to analyze the prevalence of MAFEs across progressive CKD stages.

### Functional mobility deficits study design

The second study involved a prospective cohort study of participants with DPN. The main focus of our analysis was the participants with DPN and various stages of CKD to determine the impact of CKD on the performance of functional mobility tasks. Participants were recruited from the Washington University School of Medicine Diabetes Clinic, Washington University’s Volunteers for Health, the Center for Community Based Research, and the surrounding St. Louis Community. Study inclusion criteria included individuals with a diagnosis of diabetes mellitus and evidence of PN. DM status was based on subjects’ report of a diagnosis of DM from a physician, confirmation of medication usage for DM (e.g., insulin, oral hypoglycemic agents, or both), and verification of glycated hemoglobin (A1C) levels. Participants were excluded from the study if they presented with any acute illness or hospitalization within the past 6 months, had any active infection, had prior botulinum toxin injections, or major lower extremity amputation (transtibial or transfemoral), had any serious health (e.g., stroke, Parkinson’s Disease) comorbidity, or had or were taking medication that would limit participation in physical-activity testing (e.g., congestive heart failure, angina pectoris). All participants gave their signed informed consents based on the protocol that was approved by the Human Research Protection Office’s institutional review board at Washington University School of Medicine in St. Louis, Missouri, United States (IRB ID#: 201511058). All testing took place in a single testing session.

### Assessment of peripheral neuropathy

The presence or absence of PN in each participant was assessed through light touch (pressure) at 7 locations (great toe, metatarsal heads 1 through 5, and the heel) on non-callused plantar surfaces of each foot using multiple thicknesses of Semmes Weinstein monofilaments at participant’s initial clinical visit^[14]^. Each participant was asked to shield or close their eyes from viewing their feet. The monofilament was depressed against the plantar surface of the foot just hard enough to cause a single noticeable bend in the monofilament. The participant was asked to respond yes every time the monofilament was applied. The monofilament was applied 10 times at each location and the participant was given time to respond to each pressure/light touch application. Participants unable to accurately feel the 5.07/10-gram filament at any single location were graded as absent protective sensation and were considered to have PN^[15]^.

### Modified physical performance test

The modified physical performance test (mPPT) was used to assess physical function and determine the classification of physical deficit. The mPPT is based on the Physical Performance Test originally described by Reuben *et al.*^[16–17]^. Tasks include lifting a book to an overhead shelf, donning and doffing a lab coat, picking up a coin from the floor, walking 50 feet, climbing 1 flight of stairs, climbing 4 flights of stairs, performing 5 sit-to-stand transfers from a 16-inch chair height, turning 360 degrees while standing, and standing balance (tested in side-by-side, semi tandem and tandem standing, as tolerated). The 9-item mPPT mimics activities of daily living and correlates well with disability and frailty^[18–19]^.

Each item is scored from 0 to 4 based on the time in seconds to complete each task^[19]^. Each task is performed twice, and the average time is used to score the task. The maximum score is 36 points. Those with DPN and CKD were stratified into frailty classifications based on mPPT scores: mild to no functional mobility or performance deficits and mild to no physical frailty (mPPT = 30 to 36); moderate functional mobility or performance deficits and moderate frailty with scores between 22 to 29); and severe functional mobility or performance deficits and severe frailty with scores of 21 or less. The functional mobility deficit scoring is consistent with the work by Reuben *et al.* using the original PPT^[16,17]^, in which a score of 29 or lower indicated a level of function below the 75th percentile of community-dwelling adults, and a score of 21 or lower indicated function below the 25th percentile of community-dwelling adults. Interrater reliability, validity with other functional assessments, and predictive validity of lack of independence and mortality have been reported previously for both the PPT and the mPPT^[20,21]^. The mPPT has been reported to have a test-retest reliability of 0.964, and individual items have a reliability of 0.51 to 0.99 and a Cronbach alpha of 0.785^[20,21]^.

### Ankle-brachial index

In a small subset of participants of the functional mobility deficits study, we assessed the ankle-brachial index (ABI) of either the right or left dorsalis pedis artery (ankle) and ipsilateral brachial artery (arm) using an appropriately sized cuff and Doppler to detect systolic blood pressure^[22]^. ABI values were considered normal, with values ≥ 0.80 and ≤ 1.30. ABI values ≤ 0.80 were deemed mild to moderate peripheral artery disease (PAD), while ABI values ≥ 1.30 were considered to have incompressible pedal vessels due to calcification.

### Statistical analyses

Statistical analyses were performed using IBM SPSS software v29 (IBM Corp., Armonk, NY, USA). Continuous variables were expressed as the mean value with its standard deviation. Categorical variables were expressed as frequencies or percentages. For the MAFE and mPPT datasets, risk ratios and 95% confidence intervals (95%CI) were calculated in separate models. For the MAFE dataset, we assessed the risk of sustaining each of the five MAFEs in each stage of CKD, with stage 1 CKD (no kidney disease) used as the reference group. Similarly, we assessed the risk of scoring moderate physical deficit (mPPT 22–29) or severe physical deficit (mPPT score < 22) on the mPPT in each stage of CKD. In addition, we evaluated risk ratios adjusted for age and body weight for each MAFE and mPPT category across CKD levels using multinomial logistical regression. The dependent variables were presence/absence of the MAFE or mPPT categorization. The independent variable was CKD stage, with age and body weight included as covariates. Stage 1 CKD was used as the reference group for all risk ratio analyses. An alpha value of 0.05 indicated significant findings.

## RESULTS

A total of 284 participants were included in this study, with 152 records from participants in the MAFE study and 132 participants from the functional mobility study. Table 1 shows the physical characteristics of all participants from the two studies combined, separated by study design and separated by CKD stage. There were differences in age across CKD stages, with stage 1 CKD participants being younger than all other stages and stage 5 younger than stage 4 participants. There were no differences in height, body weight, body mass index, sex distribution, diabetes phenotype, diabetes duration, ABIs, or percentage of HbA1c among study cohorts or stages of CKD. Significant differences in racial distribution were observed, with a larger proportion of Black Americans identified in the sample with stage 4 and 5 CKD. There were no differences in anti-diabetic medication use between the study cohorts, but there were differences in the anti-diabetic medications used across the stages of CKD. There was more exogenous insulin use and less OHA use in the severe stages of CKD compared to the earlier stages of CKD. Table 2 shows the frequency, unadjusted risk ratios, and 95% confidence intervals (95%CI) for each MAFE and the presence of moderate or severe mPPT scores by CKD stage. Table 3 shows the frequency and adjusted (age, body weight. race and HbA1c) risk ratios and 95%CIs for each MAFE and the presence of moderate or severe mPPT scores.

**Figure 1.**
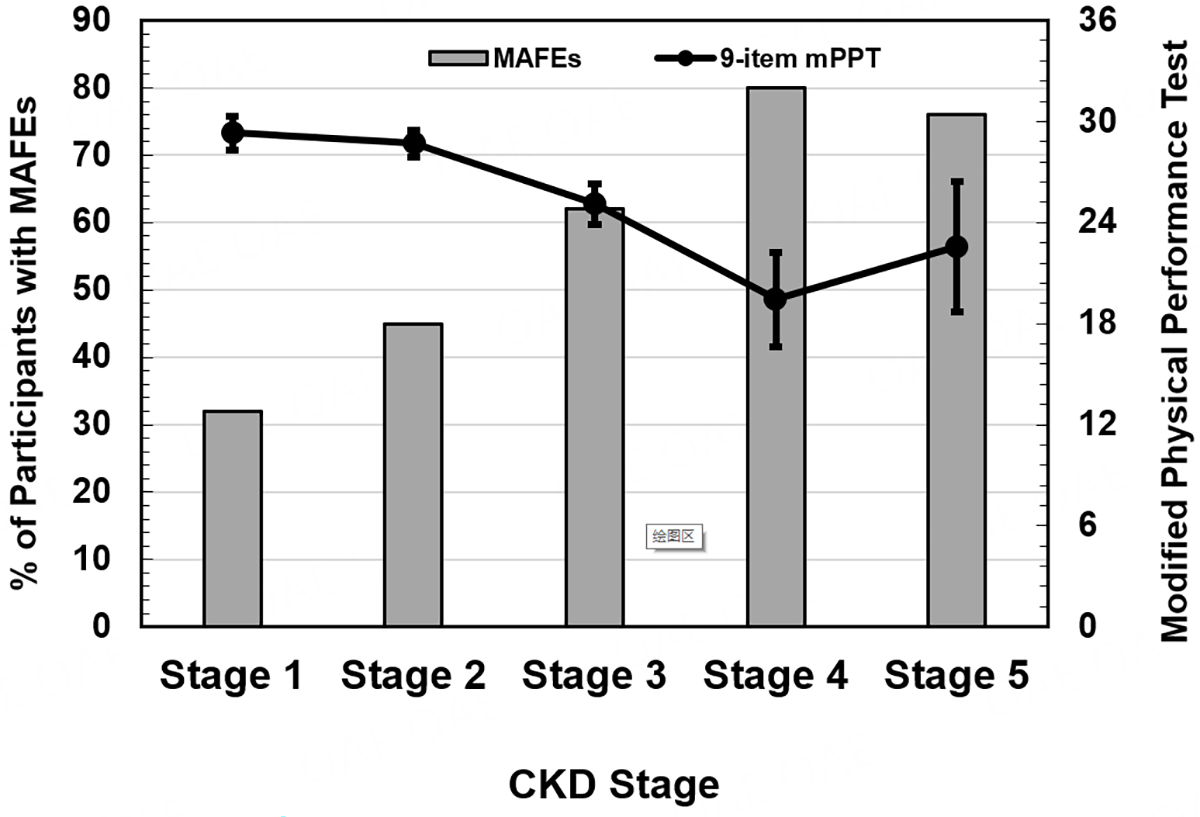
We present an inverse association between the percent of participants with at least one MAFE (primary left y-axis) and the average 9- item mPPT score (secondary right y-axis) by progressive CKD stage (x-axis). MAFE: major adverse foot events; CKD: chronic kidney disease; mPPT: modified physical performance test.

Given the absence of any participants with osteomyelitis in our sample of participants with stage 1 CKD as our reference group, we were unable to calculate unadjusted or adjusted risk ratios for this MAFE. The results indicate that the risk ratio for neuropathic foot fracture and acute Charcot neuroarthropathy remained consistent across CKD stages 2–5 compared to stage 1. The risk ratio of diabetic foot ulceration increased with more severe stages of CKD but is not statistically significant. The unadjusted risk ratio of minor amputation is significantly greater in CKD stage 5 compared to stage 1, and after adjusting for age and weight, the adjusted risk ratio was significantly greater in CKD stages 4 and 5 compared to stage 1. Regarding functional mobility deficits assessed using the mPPT, the unadjusted and adjusted risk ratios of having moderate functional mobility deficit are significantly greater with CKD stages 3 and 5 compared to stage 1. The unadjusted risk ratio of having severe physical deficit is significantly greater in CKD stages 3, 4, and 5 compared to stage 1, and the increased risk is maintained even after adjusting for age, body weight, race, and HbA1c. When combining datasets to observe potential relationships between MAFEs and physical function, we observed an inverse association between the percentage of participants with at least one MAFE and declining physical function as measured by the mPPT across CKD stages [Figure 1].

## DISCUSSION

This is the first study to determine the risks of having one or more MAFEs in those individuals with DPN across the stages of diabetic kidney disease. Similarly, this is the first study to report an increasing risk of functional mobility deficits using the standard 9-item modified physical performance test associated with a combination of DPN and progressive diabetic kidney disease.

There is a low but constant risk of foot fracture and Charcot neuroarthropathy across all the CKD stages. Unlike foot fracture and CN, MAFEs such as ulceration, osteomyelitis, and minor foot amputation or bone resection are more frequently observed in those individuals with DPN and stages 3, 4 and 5 diabetic kidney disease. Each of these MAFEs has singularly or collectively been known to lead to major lower extremity amputation (LEA)^[2–5,23]^.

The frequency of ulceration increases from 6% in stage 1 CKD (DPN without a notable decline in eGFR) to 20% and 24% of participants in stages 4 and 5, respectively. Although we are unable to calculate an accurate risk ratio of foot osteomyelitis in each stage of CKD due to an inaccurate reference group, the frequency of foot osteomyelitis in participants with Stage 4 CKD was 16% and those with Stage 5 CKD was 24%.

There is a general trend of increasing risk of amputation or bone resection when DPN combines with progressive severity of diabetic kidney disease. Previously, Margolis *et al.* reported that the hazard ratios (HRs) of incident foot ulcers in individuals with stage 3 CKD was 1.51 and stage 4 CKD was 3.22 over a median observation period of 2.4 years. Similarly, the HRs of incident LEA in stage 3 CKD was 2.28 and stage 4 was 8.05^[23]^. We report that the risk ratio increases to 25 times in those individuals with DPN and stage 5 CKD. Therefore, the combination of DPN and progressive CKD clearly raises the risk of some MAFEs and explains the observation that the highest rates of major non-traumatic LEA occur in those with end-stage renal disease^[24]^.

The findings of this study suggest that in addition to the increased risk of MAFEs as CKD and DPN progress, the risk of having moderate and severe functional mobility deficits increases. The relative risk of having a moderate functional mobility deficit is 7-fold higher by stage 3 CKD and rises to 12-fold higher by stage 5. The relative risk of severe functional mobility deficits increases 5-fold in stage 3, doubles to 10-fold by stage 4, and rises to 30-fold by stage 5. These results identify the increased frequency and relative risk of moderate and severe functional mobility deficits due to the combination of DPN and increasing stages of CKD.

The frequencies of moderate and severe functional mobility deficits in those with DPN and various stages of CKD may have been previously underestimated. As of 2022, the CDC estimates the prevalence of self-reported functional mobility deficits (e.g., walking 100 yards with aids) among individuals 60 to 74 years of age with diabetes is 19%. The estimated prevalence of self-reported difficulties with ADL and IADL among individuals 60–74 years of age with diabetes was 7% and 12%, respectively. [Diabetes Surveillance System; www.cdc.gov/diabetes/data; Division of Diabetes Translation - Centers for Disease Control and Prevention. https://gis.cdc.gov/grasp/diabetes/diabetesatlas-surveillance.html.

Fatma & Noohu compared the self-reported function of individuals with diabetes with and without peripheral neuropathy using the International Classification of Functioning, Disability and Health-core set (ICF-CS) questionnaire^[11]^. They found that 95%, 43%, and 53% of individuals with DPN reported difficulty with walking, moving around, and preparing meals, respectively, compared to individuals with diabetes but without PN (80%, 21%, and 45%, respectively)^[11]^.

Unfortunately, by relying on self-reported data, the CDC data may dramatically underestimate the prevalence of functional mobility deficits in individuals with DPN, and the Fatma & Noohu data underestimates the impact of progressive diabetic kidney disease, since our prevalence estimates of moderate and severe mobility deficits exceed 50% of individuals with both diabetic complications as early as stage 3 [Table 3]. The only previous report using the 9-item mPPT was by Tuttle *et al.*^[19]^. They reported those with DPN were 7.4 times more likely to have functional mobility deficits resulting in early frailty compared to those having diabetes without PN^[19]^.

Our result on risks may serve as a stimulus for clinicians and healthcare providers to assess the presence of DPN and closely monitor the stage of diabetic kidney disease, and take action to reduce the risks for MAFEs and accompanying functional mobility deficits. These data can serve primary care physicians, endocrinology specialists, nephrologists, podiatrists, and other rehabilitation specialists to inform their patients with diabetes of the risk of MAFEs and functional mobility deficits as early as stage 2 and stage 3 CKD. Early recognition and patient education of the increasing risks for MAFEs and functional mobility deficits may help to prevent later sequelae including major lower extremity amputation and complete disability of mobility and self-care. Future work should assess the time sequence of MAFE occurrence and further evaluate MAFE progression and physical function decline across CKD stages 2, 3a, and 3b when there may be opportunities to employ preventive interventions.

Our study has several limitations. We analyzed and combined data from two different cohort studies using different methods of data collection. The participants’ data extracted from the EMR study was retrospective and did not allow us to directly assess the mPPT score or functional mobility deficits, and there were no reports of these data in their electronic medical records. The limitation of the prospective functional mobility deficits study was that we did not record the number and types of MAFEs in each of the participants. Many participants had a history of one or more MAFEs, which may have contributed to their functional mobility deficits and mPPT scores. Despite these limitations in each cohort, study design, and data collection methods, when the participants with DPN and CKD are combined [Figure 1], there is a distinct inverse association between the number of participants with at least one or more MAFEs and the decrease in functional mobility (mPPT score) with CKD progression. These associations underscore the importance of informing patients with DPN and diabetic nephropathy of the risks of functional mobility deficits and any one or more MAFEs they could incur.

Another limitation is that we were unable to report the duration between the onset of DPN and the emergence of MAFEs and functional mobility deficits, relative to the timing of our data collection. The onset of loss of protective sensation in DPN typically occurs within 5–10 years after diagnosis of DM^[25]^, though it may vary depending on ambient blood glucose control. Though the progression of CKD from one stage to another is highly variable among individuals^[26]^, understanding the time periods of these progressions to the onset of MAFEs and functional mobility deficits may better inform clinicians and their patients of the urgency for therapeutic interventions to prevent complications.

Our study is also limited in that we did not collect a full complement of data regarding other diabetic complications (e.g., retinopathy and liver disease), cardiovascular events, or concurrent impairments including lower extremity peripheral artery diseases (PAD). PAD often accompanies diabetic complications and may have contributed to the increasing risk of MAFEs and functional mobility deficits in our two cohorts. In the small subset of participants in the functional mobility deficit study, the average ABI was not different across CKD stages, though our sample size limited any meaningful statistical comparisons [Table 1]. Though we are unable to determine the impact of PAD in our cohorts, we suspect an increasing vascular compromise will increase the risks of both MAFEs and functional mobility deficits, though this must await further study.

A last limitation is we were unable to determine the risks of MAFEs or functional mobility deficits in recently identified phenotypes of diabetic kidney disease as Di Marco *et al.* reported for cardiovascular and renal events^[10]^. We did not have a measure of urinary albumin-to-creatinine ratio (UACR) in either of our cohorts, and thus stratification of risks for MAFEs and functional mobility deficits by eGFR and UACR must await further study.

In conclusion, major adverse foot events and functional mobility deficits are prevalent in individuals with DPN and diabetic kidney disease. The risks for MAFEs and functional mobility deficits increase early in those with DPN and stage 3 CKD and increase further in those with stage 4 and 5 kidney disease.

## Figures and Tables

**Table 1.** Physical characteristics of all participants combined

	All CKD (*n* = 284)	MAFE study (*n* = 152)	mPPT study (*n* =132)	CKD stage 1 (*n* = 70)	CKD stage 2 (*n* = 89)	CKD stage 3 (*n* = 57)	CKD stage 4 (*n* = 36)	CKD stage 5 (*n* = 32)	*P*-value

Age, years	60.4 (11.5)	62.1 (11.3)	60.4 (11.5)	52.2 (10.7)	61.8 (10.5)	64.7 (10.0)	67.0 (9.9)	59.9 (10.0)	< 0.001
Sex, % male	54.2%	44.7%	65.2%	50%	59.6%	50.9%	61.1%	46.9%	0.531
Height, cm	179.2 (23.1)	165.4 (29.4)	175.7 (10.8)	174.1 (12.3)	169.9 (27.8)	168.5 (24.7)	167.1 (30.5)	168.9 (11.7)	0.558
Body weight, kg	96.9 (22.6)	92.4 (23.6)	102.1 (20.3)	100.9 (23.6)	97.2 (21.6)	97.2 (22.9)	96.9 (20.2)	86.4 (23.5)	0.057
Body mass index, kg/m^2^	32.6 (7.3)	32.2 (8.4)	33.0 (5.7)	33.4 (7.7)	32.2 (6.7)	32.9 (6.6)	33.3 (8.5)	30.3 (7.6)	0.319
T1DM, %	8%	4%	12%	9%	11%	5%	3%	9%	0.357
Duration of DM, yrs	16 (11)	15 (11)	16 (11)	13 (11)	17 (14)	15 (8)	19 (7)	19 (13)	0.097
Race									0.014
White	71.5%	73.0%	69.7%	65.7%	82.0%	78.9%	61.1%	53.1%	
Black	25.7%	23.7%	28.0%	27.1%	16.9%	19.3%	38.9%	43.8%	
Asian	0.7%	0%	1.5%	0%	1.1%	0%	0%	3.1%	
Pacific islander	0.7%	1.3%	0%	2.9%	0%	0%	0%	0%	
Unknown/ Not									
Reported	1.4%	2.0%	0.8%	4.3%	0%	1.8%	0%	0%	
HbA1c, %	7.78 (1.88)	7.75 (1.95)	7.80 (1.80)	8.3 (2.18)	7.54 (1.74)	7.67 (1.57)	7.53 (1.86)	7.76 (2.02)	0.153
Creatinine, mg/dL	1.94 (2.05)	2.13 (2.24)	1.71 (1.78)	0.77 (0.14)	0.99 (0.18)	1.54 (0.38)	2.91 (1.17)	6.74 (2.34)	< 0.001
Anti-diabetic									
Medications									
Insulin	53%	48%	59%	41%	44%	53%	71%	76%	
OHA	44%	40%	48%	55%	56%	51%	14%	9%	< 0.001
Combination	15%	18%	19%	15%	18%	15%	11%	9%	< 0.001
									0.783
ABI	NR	NR	*n* = 57	*n* = 16	*n* = 19	*n* = 13	*n* = 2	*n* = 7	
Mean (s.d.)			1.17 (0.29)	1.19 (0.18)	1.13 (0.29)	1.12 (0.30)	1.27 (0.31)	1.29 (0.52)	0.207
≥ 1.30%			27%	50%	32%	20%	50%	50%	0.778
≥ 0.79%			14%	6%	9%	27%	0%	17%	0.583

Data presented as mean (standard deviation) and percent; CKD: Chronic Kidney Disease; HbA1c: Hemoglobin A1C; T1DM: Type 1 Diabetes Mellitus; OHA: oral hypoglycemic agents including metformin, sulfonylureas; GLP-1: glucagon-like peptide 1 receptor agonists; DPP-4i: dipeptidyl peptidase IV inhibitors; TZD: thiazolidinediones; SGLT2i: sodium-glucose cotransporter 2 inhibitors; Combination: insulin and any OHA; ABI: Ankle-Brachial Index- right or left ankle (dorsalis pedis artery) to ipsilateral right or left brachial artery; NR: not recorded.

**Table 2.** Unadjusted risk ratio of major adverse foot events and physical function by stage of chronic kidney disease

	CKD stage 1	CKD stage 2	CKD stage 3	CKD stage 4	CKD stage 5

Major adverse foot events					
Fracture					
Frequency Yes/No	8/26	8/34	8/18	5/20	7/18
Risk ratio	Reference	0.81	1.31	0.85	1.19
95% confidence interval		(0.34, 1.93)	(0.57, 3.02)	(0.32, 2.29)	(0.50, 2.85)
Charcot deformity					
Frequency Yes/No	1/33	3/39	2/24	1/24	3/22
Relative risk	Reference	2.42	2.62	1.36	4.08
95% confidence interval		(0.26, 22.30)	(0.25, 27.30)	(0.09, 20.71)	(0.45, 36.95)
Ulceration					
Frequency Yes/No	2/32	4/38	4/22	5/20	6/19
Risk ratio	Reference	1.62	2.62	3.40	4.08
95% confidence interval		(0.32, 8.31)	(0.52, 13.20)	(0.72, 16.12)	(0.90, 18.56)
Osteomyelitis					
Frequency Yes/No	0/34	1/41	0/26	4/21	6/19
Relative risk	-	-	-	-	-
95% confidence interval	-	-	-	-	-
Minor amputation					
Frequency Yes/No	1/33	2/40	2/24	5/20	12/13
Risk Ratio	Reference	1.62	2.62	6.8	16.32
95% Confidence Interval		(0.15, 17.10)	(0.25, 27.30)	(0.85, 54.65)	(2.27, 117.4)
Functional mobility					
Moderate deficits (mPPT 22–29)	10/22				
Frequency Yes/No	Reference	16/23	14/9	4/2	4/1
Risk Ratio		1.31	1.95	2.13	2.56
95% Confidence Interval		(0.69, 2.48)	(1.06, 3.58)	(0.99, 4.58)	(1.30, 5.03)
Severe Deficits (mPPT < 22)	4/22				
Frequency Yes/No	Reference	6/23	8/9	5/2	2/1
Risk Ratio		1.34	3.06	4.64	4.33
95% Confidence Interval		(0.43, 4.24)	(1.09, 8.60)	(1.68, 12.8)	(1.30, 14.46)

CKD: Chronic Kidney Disease; mPPT: Modified Physical Performance Test.

**Table 3.** Age-, body weight-, Hba1c-, and race-adjusted risk ratio of major adverse foot events and physical function by stage of chronic kidney disease

	CKD stage 1	CKD stage 2	CKD stage 3	CKD stage 4	CKD stage 5

Major adverse foot event	*n* = 34	*n* = 42	*n* = 26	*n* = 25	*n* = 25
Fracture					
Frequency (%)	8 (24%)	8 (19%)	8 (31%)	5 (20%)	7 (28%)
Relative risk	Reference	0.47	0.74	0.48	0.81
95% confidence interval	-	(0.12, 1.83)	(0.16, 3.47)	(0.10, 2.24)	(0.21, 3.21)
Charcot deformity					
Frequency (%)	1 (3%)	3 (7%)	2 (8%)	1 (4%)	3 (12%)
Relative risk	Reference	1.92	2.45	1.25	4.29
95% confidence interval	-	(0.17, 21.3)	(0.17, 36.2)	(0.06, 26.2)	(0.38, 47.8)
Ulceration					
Frequency (%)	2 (6%)	4 (10%)	4 (15%)	5 (20%)	6 (24%)
Relative risk	Reference	2.33	4.34	3.66	5.04
95% confidence interval	-	(0.33, 16.57)	(0.53, 35.8)	(0.48, 28.1)	(0.80, 31.64)
Osteomyelitis					
Frequency (%)	0 (0%)	1 (2%)	0 (0%)	4 (16%)	6 (24%)
Relative risk	Reference	NA	NA	NA	NA
95% confidence interval	-	-	-	-	-
Minor amputation					
Frequency (%)	1 (3%)	2 (5%)	2 (8%)	5 (20%)	12 (48%)
Relative risk	Reference	1.48	1.33	6.18	25.04
95% confidence interval	-	(0.12, 18.8)	(0.07, 26.95)	(0.52, 73.6)	(2.72, 320.1)
Functional mobility	*n* = 32	*n* = 39	*n* = 23	*n* = 6	*n* = 5
Moderate deficits (mPPT 22–29)					
Frequency (%)	10 (31%)	16 (41%)	14 (61%)	4 (67%)	4 (80%)
Relative risk	Reference	2.61	7.03	6.79	11.7
95% confidence interval	-	(0.76, 8.95)	(1.75, 28.25)	(0.76, 61.1)	(0.96, 142.07)
	*n* = 26	*n* = 29	*n* = 17	*n* = 7	*n* = 3
Severe Deficits (mPPT < 22)					
Frequency (%)	4 (15%)	6 (21%)	8 (47%)	5 (71%)	2 (67%)
Relative risk	Reference	1.21	5.77	10.73	30.17
95% confidence interval	-	(0.20, 7.31)	(0.99, 33.76)	(0.90, 128.6)	(1.09, 833.5)

CKD: Chronic Kidney Disease; mPPT: Modified Physical Performance Test; NA: Not Applicable due to low Counts.

## Data Availability

Data will be made available upon request to the corresponding author.
